# Effect of Orexin-A on Cortisol Secretion in H295R Cells via p70S6K/4EBP1 Signaling Pathway

**DOI:** 10.1155/2015/405157

**Published:** 2015-05-10

**Authors:** Xiaocen Chang, Yuyan Zhao, Lei Guo

**Affiliations:** ^1^Department of Endocrinology, First Affiliated Hospital, China Medical University, Shenyang, Liaoning 110001, China; ^2^Department of Orthopedic Surgery, First Affiliated Hospital, China Medical University, Shenyang, Liaoning 110001, China

## Abstract

Orexin-A is a neuropeptide that orchestrates diverse central and peripheral processes. It is now clear that orexin system plays a central role in the regulation of endocrine, paracrine, and neurocrine. It is involved in the regulation of growth hormone, adrenocorticotropic hormone, thyroid, mineralocorticoid, and cortisol secretion. These hormones may also serve as a kind of signal linking energy balance regulation, reproduction, stress response, and cardiovascular regulation. Many studies have demonstrated the ability of orexin-A to regulate adrenocortical cells through the MAPK (mitogen-activated protein kinases) pathway. The aim of our study is to investigate the effect of orexin-A on cortisol secretion via the protein 70 ribosomal protein S6 kinase-1 (p70S6K) and eukaryotic translation initiation factor 4E binding proteins (4EBP1) signaling pathway in adrenocortical cells. We reported the first evidence that orexin-A stimulated p70S6K and 4EBP1 in human H295R adrenocortical cells in a concentration and time-dependent manner. 10^−6^ M orexin-A treatment for 1 hour was the most potent. Our results also indicated that p70S6K and 4EBP1 kinases participated in controlling cortisol secretion via OX_1_ receptor in H295R cells, which implied important role of p70S6K and 4EBP1 kinases in regulating adrenal function induced by orexin-A.

## 1. Introduction

Orexins, including orexin-A and orexin-B (also called hypocretin-1 and hypocretin-2), are neuropeptides discovered simultaneously in 1998 that contain 33 and 28 amino acids [1]. The actions of orexins are mediated by two membrane bound G-protein coupled receptors, orexin receptor type 1 (OX_1_ receptor) and orexin receptor type 2 (OX_2_ receptor), which display different affinity for orexins. Orexin-A is considered as a high-affinity agonist for OX_1_ receptor, whereas orexin-B has a significantly lower affinity to OX_1_ receptor. However, both peptides show similar affinities to OX_2_ receptor [2, 3]. Orexin system is widely distributed not only in the central nervous system but also in peripheral tissues [2, 4]. Great progress has been made to identify orexin system in biology and physiology over the past sixteen years. It is now clear that orexin system plays a central role in the regulation of feeding, sleeping, energy expenditure, reward seeking, and a variety of other physiological processes [4–9]. Orexins are involved in the regulation of growth hormone, adrenocorticotropic hormone, thyroid, mineralocorticoid, and cortisol secretion [5]. Orexins, mainly orexin-A, stimulate cortisol release and expression of proteins involved in steroidogenesis like steroidogenic acute regulatory protein (StAR; mRNA; and protein), different cytochrome P450 (CYP) species (mRNA), and 3*β*-hydroxysteroid dehydrogenase (HSD3B2; mRNA) [10, 11]. This indicates the orexins function on the endocrine axes.

Phospholipid kinase phosphatidylinositol 3-kinase (PI3K)/AKT/mammalian target of rapamycin (mTOR) signal pathway is an important intracellular signal transduction pathway. It plays significant role in cell apoptosis and survival by affecting the activity of downstream effector molecules, and it is closely associated with the development and progression of human tumor [12–15]. Activation of the mTOR leads to the phosphorylation and activation of downstream effectors of the pathway: the protein 70 ribosomal protein S6 kinase-1 (p70S6K) and eukaryotic translation initiation factor 4E binding proteins (4EBP1) [16]. Both p70S6K and 4EBP1 are regulators of mRNA translation and stimulate the synthesis of several proteins involved in cell growth, proliferation, cell survival, and tumorigenesis [16–19]. According to the study of Nardella et al., 25% (4 out of 16) pheochromocytomas showed very high levels of S6K1 (the p70S6K family consists of two kinases, S6K1 and S6K2). They also found that deletion of S6K1 markedly reduced the proliferation of the chromaffin cells [20]. Similarly, moderate to high staining of phosph-S6K1 and/or phosph-4EBP1 was observed in most human primary cultures of adrenocortical tumors [21]. Inhibitor treatment significantly lowered phosph-S6K1, the proliferation, and/or significantly reduced cortisol release in H295R cells [22]. These indicate the close relationship between p70S6K/4EBP1 and adrenal tumors.

It has been demonstrated that stimulation of orexin receptors may trigger activation of multiple signaling pathways, including protein kinase A (PKA), protein kinase C (PKC), and MAPK cascades-dependent mechanisms [10, 23]. Recently, researchers have begun to pay attention to the role of orexins in activation of AKT kinase along with abundant evidence indicating the key role of AKT in regulating multiple cell survival mechanisms. Sokołowska et al. found that AKT was involved in neuroprotective effects of orexins in cells subjected to chemical hypoxia [24]. In addition, Chen et al. have proved that orexin-A could affect INS-1 rat insulinoma cell proliferation and insulin secretion via AKT signaling pathway [25]. Despite AKT signaling pathway described in the literature, the role of orexin in activation of p70S6K and 4EBP1-downstream effectors of AKT/mTOR pathway is at present largely unknown. Therefore, the aim of our work was to study the effect of orexin-A on cortisol secretion via the p70S6K and 4EBP1 pathway in H295R human adrenocortical cells.

## 2. Materials and Methods

### 2.1. Reagents

The orexin-A and OX_1_ receptor antagonist SB674042 (SML0912) were obtained from Sigma (St. Louis, MO, USA). RPMI Medium 1640 and fetal bovine serum were purchased from Gibco (Grand Island, NY, USA). The mTOR inhibitor, PF-04691502, was purchased from Selleck (Houston, TX, USA). Total-p70S6K (49D7) rabbit antibody (2708), phospho-p70S6K (Thr389) (108D2) rabbit antibody (9234), total-4EBP1 (53H11) rabbit antibody (9644), and phospho-4EBP1 (Thr37/46) (236B4) rabbit antibody (2855) were obtained from Cell Signaling Technology (Danvers, MA, USA). The Cortisol Express ELISA kit was purchased from ALPCO (Paris, France).

### 2.2. Cell Culture

Human H295R adrenocortical cells were obtained from American Type Culture Collection and maintained in RPMI 1640 medium supplemented with 10% (wt/vol) fetal bovine serum, l-glutamine, penicillin (50 *μ*g/mL), and streptomycin (100 *μ*g/mL). The cells were grown in a humidified atmosphere containing 5% CO_2_ at 37°C. Before an experiment, cells (5 × 10^5^ cells/well in six-well plates) were grown in Petri dishes in serum-free medium for 24 h. The next day, cells were treated with different concentrations of orexin-A (10^−9^ M, 10^−8^ M, 10^−7^ M, and 10^−6^ M) or 10^−6^ M orexin-A with PF-04691502.

### 2.3. Cortisol Measurements

For cortisol release experiments, H295R cells were cultured in six-well plates until the cells were at about 80–85% confluence. Cells were serum-starved overnight, then washed, and incubated in fresh serum-free media containing orexin-A and/or inhibitor for 24 h. At the end of the incubation period, the supernatant was taken and snap-frozen immediately in liquid nitrogen until cortisol measurements were performed. Cortisol levels were assessed using the ELISA kit according to the manufacturer's instructions.

### 2.4. Protein Preparations and Western Blot Analysis

H295R cells were washed with cold PBS and harvested in RIPA buffer containing protease inhibitors. Cell lysates were incubated on ice for 30 min and were collected and centrifuged at 12000 g for 10 min at 4°C. The supernatants were collected and mixed with 5X loading buffer and then denatured by boiling for 10 min. Samples were separated by SDS-PAGE and transferred to PVDF membranes at 200 mA for 70 min or 45 min in a transfer buffer containing 20 mM Tris, 150 mM glycine, and 20% methanol. Membranes were incubated in nonfat dry milk for 120 min at room temperature and then washed three times with TBST for 30 min and then incubated with primary antibody against phospho/total-p70S6K at a 1 : 1000 dilution and phospho/total-4EBP1 at a 1 : 1000 dilution in TBST overnight at 4°C. The membranes were washed and incubated with a secondary antibody for 1.5 h at room temperature and then washed three times with TBST for 30 min. Protein was visualized using the ECL method. Band densities were measured using Quantity-One software.

### 2.5. Statistical Analysis

Results were expressed as a mean ± SEM and differences between the means were analyzed by one-way analysis of variance (ANOVA). *P* ≤ 0.05 was considered to be statistically significant.

## 3. Results

### 3.1. Orexin-A Stimulates the p70S6K Activity in H295R Cells

**Figure 1:**
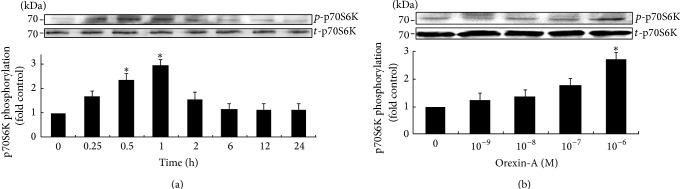
Orexin-A stimulates the p70S6K activity in H295R cells. Cells were stimulated with orexin-A (10^−6^ M) for the indicated time periods (a) or orexin-A (10^−9^ M–10^−6^ M) for 1 h (b). The expressions of p70S6K protein were measured via western blot analysis. Data are presented as mean ± SEM based on three independent experiments. Asterisk indicates significant differences as compared to control (^∗^ *P* < 0.05).

To determine the effect of orexin-A on p70S6K activity, H295R cells were stimulated with 10^−6^ M orexin-A for different periods of time. Orexin-A induced a significant increase of p70S6K phosphorylation compared with the control. The maximal phosphorylation of p70S6K (approximately 130% above the control values) was observed after 1 h of stimulation with orexin-A and then decreased, reaching 115% of the basal level after 24 h. The level of phosphorylation observed after 24 h was not statistically significant from the basal level (Figure 1(a)).

A dose-dependent study showed that orexin-A, incubated with H295R cells for 1 h, was able to activate p70S6K, with 10^−6^ M of orexin-A being the most potent (Figure 1(b)).

### 3.2. Orexin-A Stimulates the 4EBP1 Kinase Activity in H295R Cells

**Figure 2:**
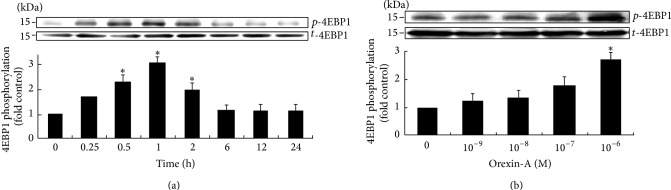
Orexin-A stimulates the 4EBP1 kinase activity in H295R cells. Cells were exposed to orexin-A at concentration of 10^−6^ M for 0.25–24 h (a). Another treatment group consisted of 10^−9^ M–10^−6^ M orexin-A for 1 h (b). The expressions of 4EBP1 protein were measured via western blot analysis. Data are presented as mean ± SEM of three independent experiments. Asterisk indicates significant differences as compared to control (^∗^ *P* < 0.05).

The effect of 4EBP1 activation by orexin-A for different time periods was analyzed by western blot. Similarly to p70S6K, orexin-A (10^−6^ M) induced a significant increase of 4EBP1 phosphorylation compared with the control, with 1 h of treatment of orexin-A being the most potent. The effect weakened between 6 and 24 h, while after 24 h of stimulation with orexin-A the phospho-4EBP1 immunoreactivity still remained higher than the basal level without statistical significance (10% above control) (Figure 2(a)).

Orexin-A treatment (10^−9^ M–10^−6^ M), for 1 h, increased 4EBP1 phosphorylation in H295R cells, and the increase was dependent upon the concentration of orexin-A, with 10^−6^ M of orexin-A being the most potent (Figure 2(b)).

### 3.3. Orexin-A Signals through the p70S6K/4EBP1 Pathways

**Figure 3:**
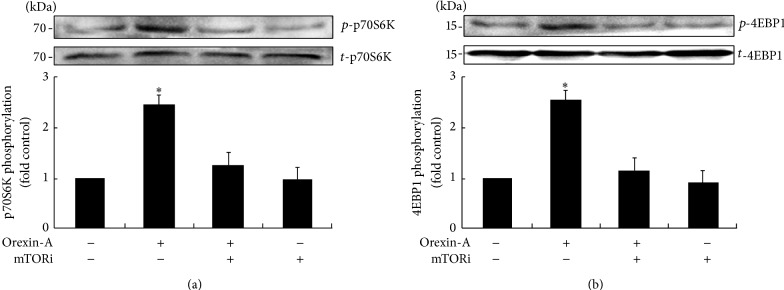
Orexin-A signals through the p70S6K/4EBP1 pathways. Cells were stimulated with orexin-A at concentration of 10^−6^ M, in the presence of PF-04691502 (10^−6^ M). Autophosphorylation of* p*-p70S6K/*p*-4EBP1 was evaluated along with the total protein activation. Protein activation was measured by western blot analysis. Data are presented as mean ± SEM of four independent experiments. Asterisks indicate significant differences compared to control samples (^∗^ *P* < 0.05).

H295R cells were exposed to orexin-A, with or without mTOR antagonist, PF-04691502. The data showed a specific increase in the p70S6K or 4EBP1 protein in H295R cells treated with 10^−6^ M orexin-A, increasing by 2.4-fold or 2.6-fold, compared to untreated controls (Figures 3(a) and 3(b)). Total-p70S6K/4EBP1 levels, however, remained unaffected by treatment. In addition, the relative increase in p70S6K/4EBP1 activation in response to orexin-A was partly abolished by the mTOR antagonist (PF-04691502, 10^−6^ M).

### 3.4. p70S6K/4EBP1 Is Involved in Orexin-A Causing Cortisol Secretion from H295R Cells

**Figure 4:**
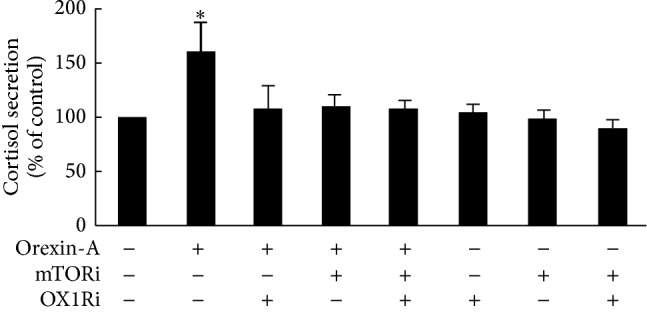
p70S6K/4EBP1 is involved in orexin-A causing cortisol secretion from H295R cells. Cells were exposed to orexin-A at concentration of 10^−6^ M for 24 h, in the presence of PF-04691502 (10^−6^ M), SB674042 (10^−6^ M), or the combination of both. Cortisol content was assessed using an ELISA kit. Data are presented as mean ± SEM of three independent experiments. Asterisks indicate significant differences compared to control (^∗^ *P* < 0.05).

To test whether the production of cortisol was affected in orexin-A-induced H295R cells, cortisol in the culture medium was assessed using the ELISA kit. The effect of orexin-A on cortisol content in the medium was determined from cell culture supernatants. The effect of 10^−6^ M orexin-A reached statistical significance, increasing cortisol secretion by 1.6-fold compared to the control. This effect disappeared in the presence of PF-04691502 (10^−6^ M), SB674042 (10^−6^ M), and the combination of both (Figure 4).

## 4. Discussion

This study demonstrates that orexin-A plays a crucial role in cortisol secretion of human H295R adrenocortical cells through the p70S6K/4EBP1 signaling pathway via OX_1_ receptor. To date, more reports have focused on the effects of orexin-A on PKA, PKC, and MAPK pathway [10, 23]. This finding shows that other signaling pathways, the p70S6K/4EBP1 pathways, also regulate the function of H295R adrenocortical cells induced by orexin-A.

In the past few years, the intracellular signaling pathways that mediate the effects of orexins have been intensively investigated. Stimulation of orexin receptors may trigger activation of classical phospholipase C (PLC) cascade, adenylate cyclase (AC), phospholipase D, phospholipase A2, and MAPK pathway [26–32]. Recently, people have begun to pay attention to the activation of AKT kinase. Previous studies from our experiment group have found that orexin-A regulates cell proliferation, apoptosis, and insulin secretion in INS-1 cells through AKT pathway [25], while the role of the neuropeptide in activation of p70S6K/4EBP1, downstream effectors of AKT/mTOR pathway, is at present largely unknown. In different types of adrenal cells, the p70S6K/4EBP1 pathway played crucial role in mediating survival signals, and altering p70S6K and/or 4EBP1 function have been associated with many pathologies. Compared with normal adrenal zona glomerulosa, the levels of phosphorylation of p70S6K were significantly upregulated in aldosterone-producing adenomas (APAs) and idiopathic hyperaldosteronism (IHA). And high staining of phospho-4EBP1 was also observed in most human primary cultures of adrenocortical tumors [33]. Given that data of Nardella et al. in mouse indicated a pivotal role for p70S6K in the proliferation of the adrenal medulla, deletion of S6K1 showed a dramatic reduction in the proliferation of the chromaffin cells [20]. Similarly, PI3K-mTOR dual inhibitor (NVP-BEZ235) was able to significantly inhibit phosphorylation of p70S6K in H295R cells and subsequently reduce proliferation in vitro and xenograft growth in vivo [22]. Therefore, the study examined the effect of orexin-A on the p70S6K/4EBP1 kinases activation in human H295R adrenocortical cells.

In in vitro experiments, many studies have shown that 10^−8^ M–10^−6^ M orexins regulate the viability, proliferation, and apoptosis of adrenal cells [10, 23]. Orexin system is widely distributed not only in the central nervous system but also in peripheral tissues, such as hypothalamus, midbrain, pancreas, and adrenal gland [2, 4]. However, immunohistochemical methods led to the identification of orexins in the bloodstream [34]. According to the report, Arihara et al. measured basal plasma orexin-A concentrations of 1.94 ± 0.24 pmol L^−1^ (corresponding to 6.89 ± 0.85 pg mL^−1^) in 17 healthy individuals, while plasma concentrations of orexin-A are differently affected by energy status and body composition [35]. To investigate the effect of orexin-A on p70S6K/4EBP1 kinases activity, H295R cells were stimulated with 10^−6^ M orexin-A for different time periods. We found that orexin-A-mediated p70S6K response was rather slow as there was no statistically significant increase of p70S6K activity during the first 15 min of incubation. The most pronounced response was observed after one hour of incubation with orexin-A. Interestingly, elevated phosphorylation-p70S6K immunoreactivity was also observed after 24 h. Orexin-A treatment for 1 h increased p70S6K phosphorylation in H295R cells, and the increase was dependent upon the concentration of orexin-A. 10^−6^ M of orexin-A led to statistically significant increase in the phosphorylation of p70S6K of H295R cells. Strikingly, the role of orexin-A on 4EBP1 kinases activity was consistent with the effect on p70S6K, while these effects disappeared in the presence of PF-04691502 (10^−6^ M). The essential role of p70S6K/4EBP1 signaling in adrenal cells survival and development prompted us to check whether these kinases could mediate the orexin-regulated cortisol secretion. In this experiment, orexin-A stimulated cortisol secretion in H295R cells, while the effect was partly abolished in the presence of mTOR inhibitor PF-04691502, OX_1_ receptor-specific antagonist of SB674042, and the combination of both.

Although the knowledge of the orexin-regulated adrenal cells is evolving, there are still many questions concerning factors mediating adrenal cells function. Results from the present study point at important role of p70S6K/4EBP1 as possible mediators of the cortisol secretion effects of orexin-A in H295R cells, while more comprehensive and specific mechanisms remain to be elucidated. We have provided the first evidence of orexin-A regulating human H295R adrenocortical cells via the p70S6K/4EBP1 signaling pathway. Together with further research utilizing p70S6K/4EBP1 inhibitors, we may provide a new and promising target in studies on diseases associated with orexin in the adrenal gland.

## References

[1] de Lecea L., Kilduff T. S., Peyron C. (1998). The hypocretins: hypothalamus-specific peptides with neuroexcitatory activity. *Proceedings of the National Academy of Sciences of the United States of America*.

[2] Sakurai T., Amemiya A., Ishii M. (1998). Orexins and orexin receptors: a family of hypothalamic neuropeptides and G protein-coupled receptors that regulate feeding behavior. *Cell*.

[3] Ammoun S., Holmqvist T., Shariatmadari R. (2003). Distinct recognition of OX1 and OX2 receptors by orexin peptides. *Journal of Pharmacology and Experimental Therapeutics*.

[4] Voisin T., Rouet-Benzineb P., Reuter N., Laburthe M. (2003). Orexins and their receptors: structural aspects and role in peripheral tissues. *Cellular and Molecular Life Sciences*.

[5] Xu T.-R., Yang Y., Ward R., Gao L., Liu Y. (2013). Orexin receptors: multi-functional therapeutic targets for sleeping disorders, eating disorders, drug addiction, cancers and other physiological disorders. *Cellular Signalling*.

[6] Malendowicz L. K., Jedrzejczak N., Belloni A. S., Trejter M., Hochól A., Nussdorfer G. G. (2001). Effects of orexins A and B on the secretory and proliferative activity of immature and regenerating rat adrenal glands. *Histology and Histopathology*.

[7] Mazzocchi G., Malendowicz L. K., Gottardo L., Aragona F., Nussdorfer G. G. (2001). Orexin A stimulates cortisol secretion from human adrenocortical cells through activation of the adenylate cyclase-dependent signaling cascade. *Journal of Clinical Endocrinology and Metabolism*.

[8] Spinazzi R., Rucinski M., Neri G., Malendowicz L. K., Nussdorfer G. G. (2005). Preproorexin and orexin receptors are expressed in cortisol-secreting adrenocortical adenomas, and orexins stimulate *in vitro* cortisol secretion and growth of tumor cells. *Journal of Clinical Endocrinology and Metabolism*.

[9] Ziolkowska A., Spinazzi R., Albertin G. (2005). Orexins stimulate glucocorticoid secretion from cultured rat and human adrenocortical cells, exclusively acting via the OX1 receptor. *Journal of Steroid Biochemistry and Molecular Biology*.

[10] Ramanjaneya M., Conner A. C., Chen J., Stanfield P. R., Randeva H. S. (2008). Orexins stimulate steroidogenic acute regulatory protein expression through multiple signaling pathways in human adrenal H295R cells. *Endocrinology*.

[11] Wenzel J., Grabinski N., Knopp C. A. (2009). Hypocretin/orexin increases the expression of steroidogenic enzymes in human adrenocortical NCI H295R cells. *American Journal of Physiology—Regulatory Integrative and Comparative Physiology*.

[12] Samuels Y., Velculescu V. E. (2004). Oncogenic mutations of PIK3CA in human cancers. *Cell Cycle*.

[13] Grozinsky-Glasberg S., Franchi G., Teng M. (2008). Octreotide and the mTOR inhibitor RAD001 (everolimus) block proliferation and interact with the Akt-mTOR-p70S6K pathway in a neuro-endocrine tumour cell line. *Neuroendocrinology*.

[14] Grozinsky-Glasberg S., Rubinfeld H., Nordenberg Y. (2010). The rapamycin-derivative RAD001 (everolimus) inhibits cell viability and interacts with the Akt-mTOR-p70S6K pathway in human medullary thyroid carcinoma cells. *Molecular and Cellular Endocrinology*.

[15] Harthill J. E., Rubio M. P., Milne F. C., MacKintosh C. (2002). Regulation of the 14-3-3-binding protein p39 by growth factors and nutrients in rat PC12 pheochromocytoma cells. *Biochemical Journal*.

[16] Guertin D. A., Sabatini D. M. (2007). Defining the role of mTOR in cancer. *Cancer Cell*.

[17] Ruggero D., Montanaro L., Ma L. (2004). The translation factor eIF-4E promotes tumor formation and cooperates with c-Myc in lymphomagenesis. *Nature Medicine*.

[18] Furic L., Rong L., Larsson O. (2010). EIF4E phosphorylation promotes tumorigenesis and is associated with prostate cancer progression. *Proceedings of the National Academy of Sciences of the United States of America*.

[19] Sonenberg N. (2008). eIF4E, the mRNA cap-binding protein: from basic discovery to translational research. *Biochemistry and Cell Biology*.

[20] Nardella C., Lunardi A., Fedele G. (2011). Differential expression of S6K2 dictates tissue-specific requirement for S6K1 in mediating aberrant mTORC1 signaling and tumorigenesis. *Cancer Research*.

[21] de Martino M. C., Feelders R. A., de Herder W. W. (2014). Characterization of the mTOR pathway in human normal adrenal and adrenocortical tumors. *Endocrine-Related Cancer*.

[22] Doghman M., Lalli E. (2012). Efficacy of the novel dual PI3-kinase/mTOR inhibitor NVP-BEZ235 in a preclinical model of adrenocortical carcinoma. *Molecular and Cellular Endocrinology*.

[23] Ramanjaneya M., Conner A. C., Chen J. (2009). Orexin-stimulated MAP kinase cascades are activated through multiple G-protein signalling pathways in human H295R adrenocortical cells: diverse roles for orexins A and B. *Journal of Endocrinology*.

[24] Sokołowska P., Urbańska A., Biegańska K. (2014). Orexins protect neuronal cell cultures against hypoxic stress: an involvement of Akt signaling. *Journal of Molecular Neuroscience*.

[25] Chen L., Zhao Y., Zheng D., Ju S., Shen Y., Guo L. (2013). Orexin A affects INS-1 rat insulinoma cell proliferation via orexin receptor 1 and the AKT signaling pathway. *International Journal of Endocrinology*.

[26] Johansson L., Ekholm M. E., Kukkonen J. P. (2007). Regulation of OX_1_ orexin/hypocretin receptor-coupling to phospholipase C by Ca^2+^ influx. *British Journal of Pharmacology*.

[27] Holmqvist T., Johansson L., Östman M., Ammoun S., Åkerman K. E. O., Kukkonen J. P. (2005). OX1 orexin receptors couple to adenylyl cyclase regulation via multiple mechanisms. *The Journal of Biological Chemistry*.

[28] Magga J., Bart G., Oker-Blom C., Kukkonen J. P., Åkerman K. E. O., Näsman J. (2006). Agonist potency differentiates G protein activation and Ca^2+^ signalling by the orexin receptor type 1. *Biochemical Pharmacology*.

[29] Tang J., Chen J., Ramanjaneya M., Punn A., Conner A. C., Randeva H. S. (2008). The signalling profile of recombinant human orexin-2 receptor. *Cellular Signalling*.

[30] Urbańska A., Sokołowska P., Woldan-Tambor A. (2012). Orexins/hypocretins acting at Gi protein-coupled OX_2_ receptors inhibit cyclic AMP synthesis in the primary neuronal cultures. *Journal of Molecular Neuroscience*.

[31] Woldan-Tambor A., Biegańska K., Wiktorowska-Owczarek A., Zawilska J. B. (2011). Activation of orexin/hypocretin type 1 receptors stimulates cAMP synthesis in primary cultures of rat astrocytes. *Pharmacological Reports*.

[32] Zhu Y., Miwa Y., Yamanaka A. (2003). Orexin receptor type-1 couples exclusively to pertussis toxin-insensitive G-proteins, while orexin receptor type-2 couples to both pertussis toxin-sensitive and -insensitive G-proteins. *Journal of Pharmacological Sciences*.

[33] Su H., Gu Y., Li F. (2013). The PI3K/AKT/mTOR signaling pathway is overactivated in primary aldosteronism. *PLoS ONE*.

[34] Heinonen M. V., Purhonen A. K., Mäkelä K. A., Herzig K. H. (2008). Functions of orexins in peripheral tissues. *Acta Physiologica*.

[35] Arihara Z., Takahashi K., Murakami O. (2001). Immunoreactive orexin-A in human plasma. *Peptides*.

